# Development and preliminary validation of Brace Questionnaire (BrQ): a new instrument for measuring quality of life of brace treated scoliotics

**DOI:** 10.1186/1748-7161-1-7

**Published:** 2006-05-20

**Authors:** Elias Vasiliadis, Theodoros B Grivas, Konstantina Gkoltsiou

**Affiliations:** 1 Orthopaedic Department, "Thriasion" General Hospital, G. Gennimata Av. 19600, Magoula, Attica, Greece; 2 2 Pediatric Department, Children's Hospital, "P.&A. Kyriakou", University of Athens Medical School, Thivon & Levadias, Goudi, 11527, Athens, Greece

## Abstract

**Background:**

The quality of life among children with idiopathic scoliosis during their adolescence has been reported to be affected by the brace itself. However, a controversy exists whether brace treated scoliotics experience a poor quality of life, thus there is a need for the development of a brace-oriented instrument, as the now-existing questionnaires that are commonly used, such as the SRS -22, take into consideration the effects of both the conservative and the surgical treatment on quality of life of scoliotic children. The aim of the present study is to assess the validity and reliability of Brace Questionnaire (BrQ), a new instrument for measuring quality of life of scoliotic adolescents who are treated conservatively with a brace.

**Material-method:**

Methodology of development involved literature review, patient and health care professionals' in-depth interviews and content validity analysis on patients. A validation study was performed on 28 brace treated scoliotic children aged between 9 and 18 years old. BrQ was assessed for the following psychometric properties: item convergent validity, floor and ceiling effects, internal consistency reliability, clinical validity and responsiveness to change.

**Results:**

BrQ is self administrated and developmentally appropriate for ages 9 to 18 years old and is consisted of 34 Likert-scale items associated with eight domains: general health perception, physical functioning, emotional functioning, self esteem and aesthetics, vitality, school activity, bodily pain and social functioning of scoliotic children treated conservatively with a brace. The subscales of these eight dimensions can be combined to produce a total score. Higher scores mean a better quality of life. An item convergent validity ≥ 0.40 was satisfied by all items in the present study. A satisfactory internal consistency reliability for the BrQ was recorded (Cronbach's alpha coefficient was 0.82). There were no floor or ceiling effects. The correlation between BrQ overall scores and mild and moderate scoliosis was statistically significant (p < 0.001), revealing high clinical validity. An increase in effect sizes for the patient with improved scoliotic curves indicates that the BrQ is responsive to change in health status.

**Conclusion:**

BrQ is reliable, valid and responsive to change in adolescents with IS who are treated conservatively with a brace.

## Background

An increased emphasis on outcome measures of various treatment methods has evolved in the recent years. There is a need to measure outcomes as a simple part of an effort to quantify and then improve the quality of health care. In order to determine the effectiveness of a specific treatment, numerous objective measures have been utilized, such as physical examination findings, radiographs and various non-validated functional scales. Quality of Life (QoL) as a multidimensional construct composed of functional, physical, emotional, social and spiritual well-being [1] has been introduced in the recent years. Traditional outcome measures are only one aspect of the overall QoL of a specific individual although there are a variety of opinions regarding the factors that contribute to QoL.

The consideration of QoL in clinical studies and various attempts to make this construct measurable to determine therapeutic success is an ongoing process [2]. It simultaneously acts as an aid for decisions on the choice of treatment strategy for chronically ill patients [3], which is obviously a challenging therapeutic aim and is at least as significant as somatic parameters [4]. QoL has therefore become a leading criterion in many outcome studies alongside physical and economic factors. In the course of this development, the concept of QoL is clearly listed as outcome parameter in many medical societies' guidelines.

Instruments that have been constructed for quantification of QoL are divided into *generic*, which are related to aspects that exist independently from any particular disease and *disease specific*, which focus on particular characteristics of specific diseases. An advantage of disease specific instruments is the precise recording of strains and limitations of a specific disease rather than those of diseases in general. The majority of current recommendations by health economists and clinical pharmacological associations include suggestions regarding the use of disease specific than generic QoL questionnaires.

AIS is considered a possible social problem and furthermore brace treatment may influence the QoL of the adolescents. There is also an increased parental concern mainly about future pain and disability as an adult [5]. Cosmetic/aesthetic results have also been an important factor to consider in the treatment of adolescent patients with scoliosis [6,7]. AIS and bracing are not associated directly with pain but they can cause discomfort and are disturbing the patient's day-life activities. In a retrospective study, 23% of 2442 patients with idiopathic scoliosis (age 6–20 years) experienced back pain [8].

Approximately 9% of girls will discontinue therapeutic brace wearing because of psychological distress related to the deformity around the hips [9]. It has been suggested that AIS may lead to multiple physical and psychosocial impairments depending on its severity [10]. Previous studies have only assessed generic health measures, functional status, body image, and self-image [10].

Braces in Adolescent Idiopathic Scoliosis (AIS) treatment are reported to produce stress [11-14] although there is a controversy whether health related QoL of brace treated scoliotics is negatively affected [10,13,15-17]. AIS is a chronic condition that affects the body configuration of the adolescent leading to certain alterations in lifestyle as a consequence. The impact of the brace to the self and body image of the adolescent is reported as the main contributory factor for stress production [18-25].

Although there are a few questionnaires for patients with AIS, a disease-specific questionnaire for brace treated children does not exist. There is a need for the development of a new brace-oriented instrument, as the questionnaires that are commonly used, such as the SRS -22, take into consideration the effects of the surgical treatment on QoL of scoliotic children. The new instrument would provide the physician a valid way to measure outcomes in order to apply the most appropriate care and to satisfy the patient's expectations in regard to the problems that the brace might produce on him or her. The aim of the present study is to assess the validity and reliability of Brace Questionnaire (BrQ), a new instrument for measuring QoL of scoliotic children who are treated conservatively with a brace.

## Method and material

### The questionnaire

The development was initiated by thorough literature review and by the formation of potential brace-related items on the basis of the results of a series of patient and health care professionals' (psychologists, social workers, health visitors, orthotics and orthopaedic surgeons) in-depth interviews.

The questions were collected and the most appropriate ones were selected by the authors and then they were grouped to eight specific domains, namely a) general health perception, b) physical functioning, c) emotional functioning, d) self-esteem and aesthetics, e) vitality, f) school activity, g) bodily pain and h) social functioning, Table 1 It was ensured that all the items chosen were consistent with the need-based theory of QoL [26]. Furthermore the items were formulated so that they could be meaningfully answered with the following five response categories: "Always", "Most of the time", "Sometimes", "Almost Never" and "Never". The procedure of selection which in fact was a first step of item reduction involved the exclusion of questions that were not relevant to QoL or items that were covering similar themes.

**Table 1:** The BrQ domains and the results of tests of item convergent validity, item consistency reliability and floor and ceiling effects for each domain of the BrQ

**BrQ Domains**	**Number of items**	**Item convergent validity^a^**	**Internal consistency reliability^b^**	**Floor effects^c^**	**Ceiling effects^d^**
General health perception	2	100%	0.72	0 (0.0%)	1 (3.6%)
Physical functioning	7	100%	0.80	0 (0.0%)	0 (0.0%)
Emotional functioning	5	100%	0.77	0 (0.0%)	0 (0.0%)
Self esteem and aesthetics	2	100%	0.88	0 (0.0%)	0 (0.0%)
Vitality	2	100%	0.84	0 (0.0%)	0 (0.0%)
School activity	3	100%	0.82	0 (0.0%)	3 (10.7%)
Bodily pain	6	100%	0.85	0 (0.0%)	2 (7.1%)
Social functioning	7	100%	0.88	0 (0.0%)	3 (10.7%)

^a ^Percentage of item-scale correlations ≥ 0.40.

^b ^Cronbach's alpha coefficient

^c ^Percentage of respondents with minimum scale scores

^d ^Percentage of respondents with maximum scale scores

### The studied population

A validation study was performed in order to reduce the number of items through psychometric analysis and to perform content validity of the questionnaire on patients. The validation study involved 28 scoliotic children conservatively treated with a Dynamic Derotation Brace, a modified Boston brace with antirotatory blades [27]. All the 28 children are followed at the Scoliosis Clinic of the Orthopaedic Department of Thriasio General Hospital of Athens, Greece. Seventeen out of 28 children had right thoracic and left lumbar curves with a mean age 13.3 years (range 9–17), 15 girls and 2 boys with a mean thoracic Cobb angle 23.2° (range 10°–38°) and a mean lumbar 21.2° (range 8°–36°) respectively. Rotation was measured at a mean value of 6.9° (range 3°–25°) for the thoracic curve and 7.8° (range 4°–15°) for the lumbar curve. Four out of 28 children (all girls) had right thoracic curves with a mean age 13.8 years (range 12–15 years). Mean thoracic Cobb angle was 25° (range 22°–35°) and a mean apical vertebral rotation 6.8° (range 3°–10°). Seven out of 28 children (6 girls and 1 boy) had thoracolumbar curves with a mean age 13.5 years (range 12–18 years), with a mean Cobb angle 24° (range 20°–38°) and a mean apical vertebral rotation 10° (range 4°–30°).

### Psychometric evaluation

The BrQ was assessed for the following psychometric properties: item convergent validity (item-scale correlations should be ≥ 0.4) [28], floor and ceiling effects (the percentage scoring the lowest and highest possible scores), internal consistency reliability (estimates how consistently individuals respond to the items within a scale and is measured with Cronbach's alpha), clinical validity and responsiveness to change.

The clinical validity of the BrQ was assessed by describing and comparing BrQ overall scores of the children according to the severity of their scoliosis. The patients were divided to mild and moderate subgroups if their major curve was below or higher than 30° respectively. Fifteen patients were in the mild and 13 were in the moderate subgroup. The hypothesis was that patients with more severe curves would have worse QoL, indicated by lower BrQ overall scores.

The responsiveness of the BrQ to change over time was assessed by comparing BrQ overall change scores among patients defined as improved, stable and deteriorated from baseline to re-evaluation during the follow up period, on the basis of their curve change. The criterion for improvement or deteriorating of a curve was a Cobb angle change > 5° compared with the initial reading on the radiographs [29] with the child out of brace. Re-evaluation was performed at least 2 years after the initiation of brace treatment. The BrQ was filled always prior to clinical evaluation or any discussion with the physician.

The effect size (ES) was used as a measure of the change in BrQ overall scores within each group. Effect sizes were calculated by dividing the change in mean BrQ overall scores by the standard deviation of mean scores at baseline. The ES has been recommended in the literature as an appropriate point of reference for evaluating the magnitude and meaning of change in health status measures [30].

### Statistical analysis

Kolmogorov-Smirnov goodness-of-fit test and Shapiro-Wilk test for normality did not find the analysis data to be normally distributed; therefore non-parametric tests were used for the statistical analysis. Pearson's correlation coefficient was used for all correlations evaluated; the Kruskall-Wallis test was used for comparisons between more than two groups; the Mann-Whitney-Wilcoxon test was used for comparisons between pairs of groups and the Wilcoxon signed rank test was used for comparing two points in time within groups. P values of less than 0.05 were considered to be significant. Internal consistency was evaluated by Cronbach's alpha method.

## Results

### The questionnaire

A final 34 Likert scale items questionnaire was constructed. The questionnaire was designed to be self administrated and developmentally appropriate for ages 9 to 18 years old. Administration of the BrQ lasted 10–12 minutes. The English translation of the resulting 34 items of the BrQ is provided in **Appendix 1**, Additional file 1.

Scoring of BrQ is simple. For items 4, 5, 6, 12, 14, 15, 16 and 17 "Always" received a score of 5, "Most of the time" received a score of 4, "Sometimes" received a score of 3, "Almost Never" received a score of 2 and "Never" received a score of 1. For items 1, 2, 3, 7, 8, 9, 10, 11, 13, 18, 19, 20, 21, 22, 23, 24, 25, 26, 27, 28, 29, 30, 31, 32, 33 and 34 "Always" received a score of 1, "Most of the time" received a score of 2, "Sometimes" received a score of 3, "Almost Never" received a score of 4 and "Never" received a score of 5. Each item score is then multiplied by 20 and the total score is divided by 34. The minimum score is theoretically 20 and the maximum is 100. A higher score indicates better QoL. A subscale score can be calculated for each of the eight domains by means of dividing the total score of each dimension divided by the number of items that comprise it. A computer program has been developed for the calculation of the overall and the subscales' score of the BrQ.

### Factor analysis

The results of the content validity analysis demonstrated excellent reliability and content validity for the BrQ, as summarized in Table 1.

### Item convergent validity

The criterion for item convergent validity (item-scale correlations ≥ 0.40) was satisfied by all items in the present study. The item convergent validity for each domain of the BrQ is shown in Table 1

### Internal consistency reliability

Cronbach's alpha coefficients for the BrQ overall score were 0.82, exceeding the minimum recommended standard of 0.70 and indicating satisfactory internal consistency reliability for the BrQ. Cronbach's alpha coefficients for each BrQ domain is shown in Table 1

### Floor and ceiling effects for the BrQ overall score

For the BrQ overall score, in the present study, 0 % of patients scored at floor and 0% scored at ceiling. Therefore, there were no floor or ceiling effects for the BrQ overall score. Floor and ceiling effects for each domain of the BrQ are shown in Table 1 Floor and ceiling effects for each item are shown in Table 2

**Table 2:** Floor and ceiling effects (percentage of respondents with minimum/maximum scale scores) for each item of the BrQ

**No of Item**	**Floor effect**	**Ceiling effect**
1	1 (3.58%)	2 (7.14%)
2	0 (0.00%)	2 (7.14%)
3	2 (7.14%)	0 (0.00%)
4	2 (7.14%)	1 (3.58%)
5	3 (10.71%)	0 (0.00%)
6	1 (3.58%)	2 (7.14%)
7	1 (3.58%)	1 (3.58%)
8	0 (0.00%)	2 (7.14%)
9	1 (3.58%)	2 (7.14%)
10	1 (3.58%)	1 (3.58%)
11	1 (3.58%)	1 (3.58%)
12	1 (3.58%)	0 (0.00%)
13	0 (0.00%)	2 (7.14%)
14	0 (0.00%)	3 (10.71%)
15	1 (3.58%)	1 (3.58%)
16	1 (3.58%)	0 (0.00%)
17	0 (0.00%)	0 (0.00%)
18	0 (0.00%)	0 (0.00%)
19	0 (0.00%)	5 (17.86%)
20	1 (3.58%)	4 (14.29%)
21	0 (0.00%)	3 (10.71%)
22	0 (0.00%)	4 (14.29%)
23	1 (3.58%)	3 (10.71%)
24	1 (3.58%)	3 (10.71%)
25	0 (0.00%)	0 (0.00%)
26	0 (0.00%)	0 (0.00%)
27	1 (3.58%)	2 (7.14%)
28	1 (3.58%)	3 (10.71%)
29	1 (3.58%)	4 (14.29%)
30	1 (3.58%)	3 (10.71%)
31	1 (3.58%)	5 (17.86%)
32	0 (0.00%)	4 (14.29%)
33	1 (3.58%)	3 (10.71%)
34	2 (7.14%)	1 (3.58%)

### Clinical validity

**Figure 1:**
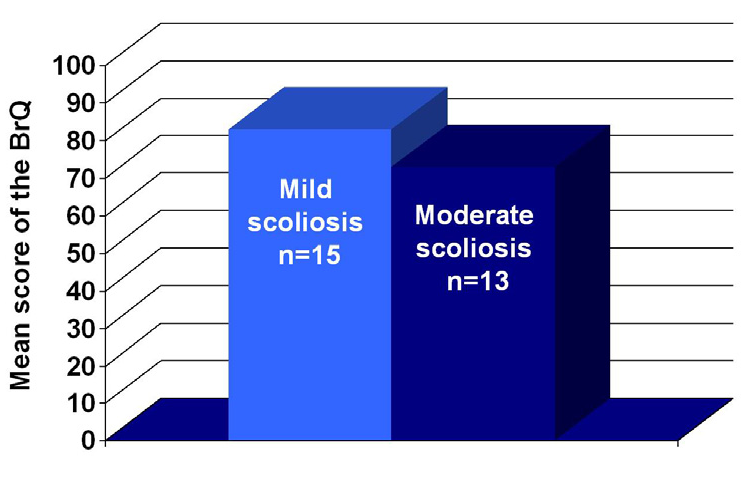
Clinical validity. Comparison of BrQ overall scores for mild and moderate scoliosis subgroups (see text). p < 0.001 (Mann-Whitney-Wilcoxon test comparing the two subgroups).

Clinical validity was assessed by examining the correlation between BrQ overall scores and severity of the scoliotic curve. Fifteen patients with a mean age of 13 years old (9–17 years old) and a mean Cobb angle of 20.2° (18° -29°) were in the mild subgroup and 13 patients with a mean age of 13.9 years old (12–18 years old) and a mean Cobb angle of 32.4° (30° -38°) were in the moderate subgroup. The correlation between BrQ overall scores for mild and moderate scoliosis was statistically significant (p < 0.001), Figure 1 Impairment in QoL due to the brace was greater for subgroups with greater scoliotic curves. The differences between pairs of adjacent subgroups were assessed further using the Mann-Whitney-Wilcoxon test. Nevertheless, statistically significant differences in BrQ overall scores between the two subgroups, <30° and >30°, was observed (p < 0.001). These findings provide evidence that the BrQ is clinically valid in this clinical trial population, Table 1

### Responsiveness to change over time

**Figure 2:**
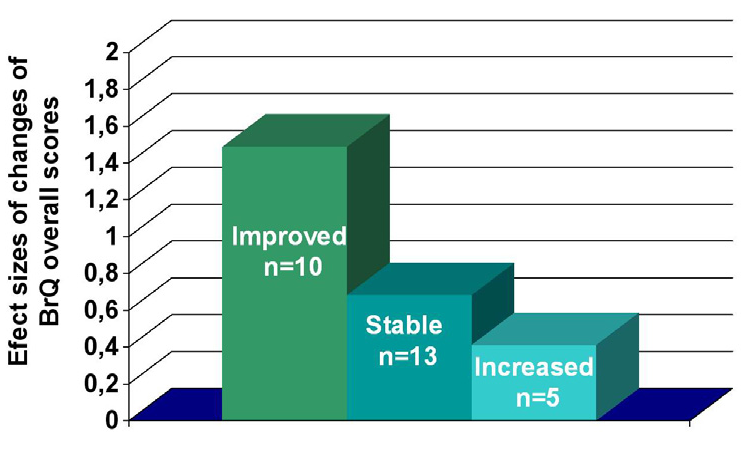
Responsiveness. Effect sizes as a measure of the change in BrQ overall change scores among patient with improved, stable and increased curves during the follow up period (see text). p < 0.001 for comparisons of the change in BrQ overall scores among the three subgroups (Kruskall-Wallis test).

Ten patients (35%) with a mean age of 12,9 years old (12–17 years old) and a mean Cobb angle of 17,1° (12° -25°) were improved, 13 patients (46%) with a mean age of 13,4 years old (12–18 years old) and a mean Cobb angle of 24,2° (20° -36°) were stable and 5 patients (18%) with a mean age of 14,5 years old (9–16 years old) and a mean Cobb angle of 32° (26° -38°) were deteriorated. The responsiveness of the BrQ to change over time was suggested by moderate and statistically significant correlations of BrQ overall change scores among patient with improved, stable and increased curves during the follow up period, Figure 2 A step-wise increase in effect sizes for the improved, stable and deteriorated subgroup indicating greater improvements in BrQ overall scores for the more improved subgroups compared with the stable and deteriorated subgroups. ES has indicated large improvements in overall scores in the improved subgroup (ES = 1.49) moderate improvements in the stable subgroup (ES = 0.68) and small improvements in the deteriorated subgroup (ES = 0.41), Table 3 These findings indicate that the BrQ is responsive to clinician-rated changes in health status.

**Table 3:** Changes in BrQ overall scores among patients defined as improved, stable and deteriorated from baseline to re-evaluation during the follow up period, at least 2 years after the initiation of brace treatment on the basis of their curve change. Effect sizes were calculated by dividing the change in mean score by the standard deviation of the first mean score of the BrQ.

**Subgroups according to the curve change**	**n**	**First BrQ overall score mean**	**First Standard Deviation (total)**	**Follow up BrQ overall score mean**	**Mean score change**	**Effect Size**
Improved	10	64	14.77	86	22	1.49
Stable	13	61	14.77	71	10	0.68
Increased	5	60	14.77	66	6	0.41

### Missing data

Missing data for each of the items of the BrQ ranged from 0% missing data for items 1–10, 12–28, 30–34 to 3.57% (n = 1) for items 11 ("You felt worried because of the brace") and 29 ("Your friends felt compassion for you"). Therefore, missing data at the item level were not problematic.

## Discussion

Beyond definition, quality in health care is determined by the application of the right method of care to the patient's condition in the most effective manner possible and by the nature of the interaction between the patient and the provider [31]. This subjective aspect of quality is the portion of the outcome measures that BrQ is attempting to quantify.

Climent and Sanchez in their study of adolescents with spinal deformities contended that QoL variables include the Risser sign, clinical diagnosis, duration of brace treatment, and degree of correction [32]. These variables do not constitute a significant measurement of patient wellbeing, are more related to the diagnostic evaluation and do nothing to alter one's perception of happiness. Health educators, school nurses, and clinicians need to be aware of social well-being factors, and how these factors relate to psychosocial functioning [33].

In order to evaluate the effectiveness of brace treatment, we need to determine three major factors, namely the patient, the multidisciplinary team that provides care and the brace itself. Although it would seem intuitive that a *patient's *physical, emotional, and social well-being would all have a powerful effect on his or her ability to benefit from brace treatment, there has been little research on this important determinant of brace effectiveness. The conservative treatment that is provided by the team of professionals is the *method*. The brace is the *mean*. There are very few data indicating that improvements in the type of the brace have a significant effect on the patient's QoL.

In this study we have described the development and preliminary validation of the BrQ, a questionnaire to measure the effect of brace in conservatively treated children with AIS. To our knowledge this is the first questionnaire specifically developed and validated to measure clinical success in the management of AIS patients with a brace. The items were generated by literature search and interviewing clinicians and patients. Items were thereafter selected on their clinical importance and were grouped to eight domains.

It could be argued that a possible bias was present in the item selection phase. While we cannot rule out that some kind of biased selection of items may have been present during one or more steps of the development of the questionnaire, we find it important to underline that in our view selection of items is always a qualitative process, and thus somehow subjective. However, the resulting instrument has been subjected to a quantitative analysis based on classical test theory approaches with fairly acceptable results [34].

A specific instrument such as BrQ has its self-evident strengths as compared with generic instruments by virtue of its increased sensitivity to the unique problems related to the brace itself.

Minimal important differences or minimal important change over time were not examined for the BrQ scales. Knowledge of minimal important differences are important for interpreting the meaning of health related QoL results, thus, in future studies some attempt should be made to define minimal important differences for the BrQ.

The internal consistency reliability of items in the BrQ overall score was acceptable, with Cronbach's coefficients exceeding the accepted standard (≥ 0.70). There were no floor or ceiling effects in the present study.

BrQ overall score was able to distinguish between patients with mild and moderate scoliosis. The results indicate that patients with moderate scoliosis also had lower BrQ overall scores (poorer QoL). Adolescents with severe scoliosis were excluded from the study because these patients are considered potential candidates for surgical correction, thus other specific instruments such as SRS 22 would be applied [35].

The responsiveness of the BrQ to change over time was confirmed by comparing change scores from two different measurements, at the initiation of treatment and at follow up. Correlation of changes in BrQ overall score in patients with improved, stable and increased curves was statistically significant. BrQ overall change scores were able to distinguish between these subgroups at a statistically significant level. ES's indicated the improvements were always greater in those patients rated as "improved" but only small or moderate in those rated as "stable". Sample sizes for the three groups were small and results for these subgroups should be interpreted with caution. The sensitivity of the BrQ to change could not be fully evaluated in this study owing to the small sample size and should be investigated further in a larger sample of patients. Therefore, responsiveness to change of the BrQ will be assessed with further research.

## Conclusion

In conclusion, the BrQ is reliable, valid and responsive to change in children with AIS who are treated conservatively with a brace. BrQ takes only 10 minutes to complete and covers most of the aspects of life affected by the brace.

## Authors' contributions

EV built the structure of the BrQ, was responsible for the methodological setting of the study, performed the statistical analysis and has written most parts of the manuscript. TBG was the clinical supervisor, conceived the idea of creating BrQ, designed the study, performed part of literature review and revised the manuscript for important intellectual content. KG performed part of literature review, conducted analyses, built the structure of the BrQ and contributed to the manuscript drafting. All authors have read and approved the final manuscript.

## Supplementary Material

Additional File 1 The Brace Questionnaire The English translation of the BrQ items. BrQ is validated only in its original Greek version. Click here for file
