# Supplementary material for: Development and preliminary validation of Brace Questionnaire (BrQ): a new instrument for measuring quality of life of brace treated scoliotics
Source: Scoliosis. 2006 May 20;1:7. doi: 10.1186/1748-7161-1-7 (PMC1481574; doi:10.1186/1748-7161-1-7)
Supplement: Additional File 1 — The Brace Questionnaire The English translation of the BrQ items. BrQ is validated only in its original Greek version. [file 1748-7161-1-7-S1.pdf]

BRACE QUESTIONNAIRE

This questionnaire asks how you feel about your health, while you are wearing a brace. This is not a test and there are no right or wrong answers

- ***Please read carefully every question***
- ***Choose the best answer and mark with an x***

**Example**	**Never**	**Almost never**	**Sometimes**	**Most of the time**	**Always**
**During the last week you were in a good mood for studying**	☐	☐	☐	x	☐

Please tell us a few things about yourself:

You are: ☐ a girl ☐ a boy Age: ……… years old

You are wearing the brace since ………

You are wearing the brace for ….. hours /day

Date...

During the past 3 months...	Never	Almost never	Sometimes	Most of the time	Always
1. The brace made you feel ill	☐	☐	☐	☐	☐
2. You were afraid that your back will get worse	☐	☐	☐	☐	☐

During the past 3 months while you were wearing the brace...	Never	Almost never	Sometimes	Most of the time	Always
3. You felt tired when walking	☐	☐	☐	☐	☐
4. You were able to run	☐	☐	☐	☐	☐
5. You managed to wear the brace without any help	☐	☐	☐	☐	☐
6. You managed to take out the brace without any help	☐	☐	☐	☐	☐
7. You couldn’t eat well	☐	☐	☐	☐	☐
8. You couldn’t sleep well	☐	☐	☐	☐	☐
9. You couldn’t breath well	☐	☐	☐	☐	☐

During the past 3 months...	Never	Almost never	Sometimes	Most of the time	Always
**10. The brace made you feel nervous**	☐	☐	☐	☐	☐
**11. You felt worried because of the brace**	☐	☐	☐	☐	☐
**12. You felt happy**	☐	☐	☐	☐	☐
**13. You believed that your life would be better if you were not on brace**	☐	☐	☐	☐	☐
**14. You believed that brace treatment was beneficial**	☐	☐	☐	☐	☐

During the past 1 month...	Never	Almost never	Sometimes	Most of the time	Always
**15. You felt proud of yourself**	☐	☐	☐	☐	☐
**16. You were satisfied with your body**	☐	☐	☐	☐	☐

During the past 1 month...	Never	Almost never	Sometimes	Most of the time	Always
**17. You felt strong and full of energy**	☐	☐	☐	☐	☐
**18. You felt tired and exhausted because of the brace**	☐	☐	☐	☐	☐

During the past 1 month, because of the brace...	Never	Almost never	Sometimes	Most of the time	Always
**19. You had difficulties with your lessons**	☐	☐	☐	☐	☐
**20. You were absent from school**	☐	☐	☐	☐	☐
**21. You found it hard to pay attention in the classroom**	☐	☐	☐	☐	☐

During the past 1 month, while you were wearing the brace...	Never	Almost never	Sometimes	Most of the time	Always
**22. You had to take medication for pain**	☐	☐	☐	☐	☐
**23. You had pain during the night**	☐	☐	☐	☐	☐
**24. You had pain when walking**	☐	☐	☐	☐	☐
**25. You had pain when sitting**	☐	☐	☐	☐	☐
**26. You had pain when climbing stairs**	☐	☐	☐	☐	☐
**27. You felt pins and needles to your arms or legs**	☐	☐	☐	☐	☐

During the past 1 month, because of the brace...	**Never**	**Almost never**	**Sometimes**	**Most of the time**	**Always**
**28. You couldn't go out with your friends**	☐	☐	☐	☐	☐
**29. Your friends felt compassion for you**	☐	☐	☐	☐	☐
**30. You felt different from your peers**	☐	☐	☐	☐	☐
**31. You had problems with your family**	☐	☐	☐	☐	☐
**32. You believed that your relationship with your family or your friends would be better if you were not on brace**	☐	☐	☐	☐	☐
**33. You stayed at home because you were ashamed**	☐	☐	☐	☐	☐
**34. You worn special clothes**	☐	☐	☐	☐	☐

Thank you!
